# How gibberellin-related compounds shape far-red light responses in *Marchantia polymorpha*

**DOI:** 10.1093/plcell/koad222

**Published:** 2023-08-18

**Authors:** Arpita Yadav

**Affiliations:** Assistant Features Editor, The Plant Cell, American Society of Plant Biologists; Biology Department, University of Massachusetts Amherst, MA 01003, USA

Plants have evolved diverse methods for sensing and responding to changes in their light environment. Far-red (FR) light is an important environmental signal that triggers specific developmental responses in plants, such as the hyponastic growth of petioles. Gibberellic acid (GA) biosynthesis is frequently induced during this process, contributing to petiole elongation. Although the function of GAs in mediating light responses has been thoroughly investigated in angiosperms, its conservation across land plants is unclear. The presence of this pathway in extant bryophytes, such as the liverwort *Marchantia polymorpha*, would support the hypothesis that the function of GA in mediating light response has been conserved since the most recent common ancestor of the vascular and nonvascular plants that lived on lands more than 400 million years ago.

In this issue, **Rui Sun and colleagues** (Sun et al. 2023) investigated the impact of gibberellin-related compounds on the response of *M. polymorpha* to FR light. Previous genome-wide investigations in *M. polymorpha* led to the identification of homologs of key enzymes involved in GA biosynthesis, including ent-copalyl diphosphate synthase (CPS), ent-kaurene synthase (KS), ent-kaurene oxidase, and ent-kaurenoic acid oxidase (Kumar et al. 2016; Bowman et al. 2017). These enzymes play a crucial role in the transformation of geranylgeranyl diphosphate into ent-kaurene (KA), the precursor for biologically active GAs. The existence of these homologous genes suggests the possibility of GA production in this liverwort species. Transcriptome analysis by Briginshaw et al. (2022) revealed that upregulation of GA biosynthesis gene homologs occurred under FR light-enriched conditions, implying a role for this pathway in the response to light quality change. To clarify the involvement of GA-related chemicals in FR light responses, Sun et al. analyzed different *M. polymorpha* mutant lines with defects in genes responsible for GA production.

Under conditions enriched with FR light, wild-type *M. polymorpha* plants displayed a growth pattern characterized by narrow and hyponastic thallus, along with the initiation of gametangiophore (sexual structures) formation. This was accompanied by an upregulation in the expression of genes involved in gibberellin biosynthesis and an accumulation of the GA precursor GA_12_. On the other hand, mutations in genes related to gibberellin biosynthesis, such as Mp*CPS*, exhibited a wide and flat thallus and a delay in the formation of gametangiophores (Fig. 1). Introducing the GA precursor KA to the Mp*cps* mutants restored the wild-type phenotype in a dose-dependent manner. Interestingly, no active GAs from angiosperms could rescue the phenotype, suggesting that the mutant phenotypes were caused by a deficiency of KA-derived bioactive diterpenoid compound(s) referred to as GA_Mp_.

**Figure 1.**
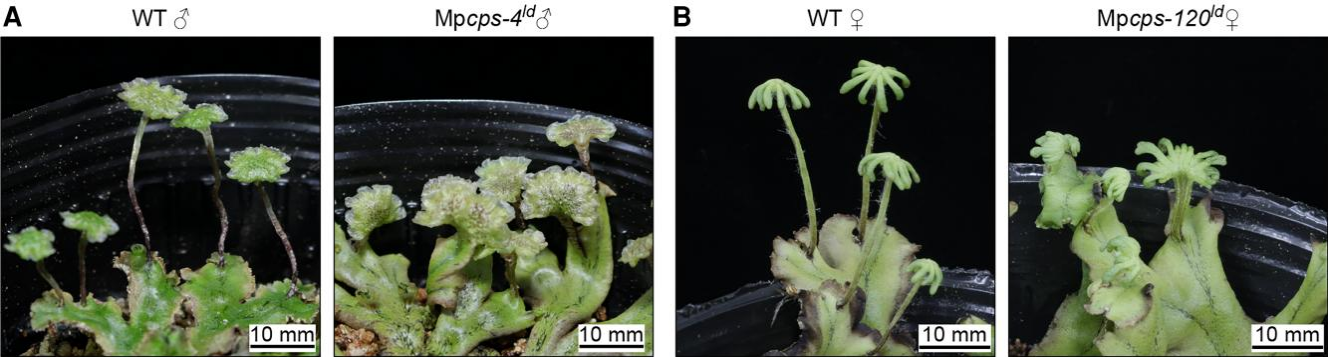
CPS is required for delayed sexual reproduction and affects gametengiophore morphology. Images of male (**A**) and female (**B**) *M. polymorpha* wild type and Mp*cps* mutants bearing gametangiophores grown under continuous white light + FR light. Adapted from Sun et al. 2023, Figure 2.

FR light-induced hyponastic growth in the thallus of *M. polymorpha* is characterized by regional growth driven by cell division in the apical meristems. This differs from shade-avoidance responses observed in angiosperms, which are primarily based on cell elongation. The authors observed that the stalks of gametangiophores in the GA_Mp_ biosynthesis mutant Mp*cps* displayed similar characteristics to Mp*kan* mutants that lack a transcription factor (MpKANADI) responsible for regulating tissue polarity (Briginshaw et al. 2022). This suggests that GA_Mp_ might also play a role in regulating tissue polarity in *M. polymorpha*. It is noteworthy that the biosynthesis of GA_Mp_ demonstrated a specific response to FR light in *M. polymorpha*. In white light, the deficiency of GA_Mp_ had negligible effects on vegetative growth, but when exposed to an enrichment of FR light, GA_Mp_ effectively suppressed thallus growth.

In the comparative analysis of GA-related diterpenoid biosynthesis in *M. polymorpha* and *Physcomitrella patens*, the authors observed conservation of components in the biosynthesis of KA. However, notable distinctions were identified in the synthesis of bioactive compounds derived from KA. GAs in vascular plants are detected through GID1 receptors. However, bryophytes do not possess these receptors, indicating distinct mechanisms for perceiving GAs and regulating responses to FR light. The hyponastic responses observed in *M. polymorpha* under FR light conditions, similar to *A. thaliana* and other land plants, are regulated by a single phytochrome (Mpphy) and a transcription factor, PHYTOCHROME-INTERACTING FACTOR (MpPIF), which is present in a single copy (Inoue et al. 2016, 2019). In this research, the authors found that the FR light-mediated biosynthesis of GA_Mp_ is also dependent on MpPIF.

Exploring the ecological relevance of gibberellin-related chemicals in the natural habitats of *M. polymorpha* could aid our understanding of how these compounds contribute to the adaptability of liverworts under varied light conditions in the wild. We can acquire a better knowledge of plant evolution and the larger implications of light-mediated developmental responses in land plants by delving deeper into the comparison of gibberellin signaling in bryophytes and vascular plants.

## Dive Curated Terms

The following phenotypic, genotypic, and functional terms are of significance to the work described in this paper:

kan Gramene: AT5G16560
kan Araport: AT5G16560

gibberellic acid CHEBI: CHEBI:86644
